# Risk analysis of the Unity 1.5T MR‐Linac adapt‐to‐shape workflow

**DOI:** 10.1002/acm2.70095

**Published:** 2025-04-16

**Authors:** Jiayi Liang, Eric Aliotta, Neelam Tyagi, Paola Godoy Scripes, Nicolas Côté, Ergys Subashi, Qijie Huang, Lian Sun, Ching‐Yun Chan, Angela Ng, Theresa Wunner, Victoria Brennan, Kaveh Zakeri, James Mechalakos

**Affiliations:** ^1^ Department of Medical Physics Memorial Sloan Kettering Cancer Center New York USA; ^2^ Department of Radiation Oncology Memorial Sloan Kettering Cancer Center New York USA

**Keywords:** adaptive treatment, adapt‐to‐shape, MR‐Linac, process map, radiation oncology, risk analysis

## Abstract

**Background and Purpose:**

The adapt‐to‐shape (ATS) workflow on the Unity MR‐Linac (Elekta AB, Stockholm, Sweden) allows for full replanning including recontouring and reoptimization^5^. Additional complexity to this workflow is added when the adaptation involves the use of MIM Maestro (MIM Software, Cleveland, OH) software in conjunction with Monaco (Elekta AB, Stockholm, Sweden). Given the interplay of various systems and the inherent complexity of the ATS workflow, a risk analysis would be instructive.

**Method:**

Failure modes and effects analysis (FMEA) following Task Group 100^13^ was completed to evaluate the ATS workflow. A multi‐disciplinary team was formed for this analysis. The team created a process map detailing the steps involved in ATS treating both the standard Monaco workflow and a workflow with the use of MIM software in parallel. From this, failure modes were identified, scored using three categories (likelihood of occurrence, severity, and detectability which multiplied create a risk priority number), and then mitigations for the top 20^th^ percentile of failure modes were found.

**Results:**

Risk analysis found 264 failure modes in the ATS workflow. Of those, 82 were high‐ranking failure modes that ranked in the top 20^th^ percentile for risk priority number and severity scores. Although high‐ranking failure modes were identified in each step in the process, 62 of them were found in the contouring and planning steps, highlighting key differences from adapt‐to‐position (ATP), where the importance of these steps are minimized. Mitigations are suggested for all high‐ranking failure modes.

**Conclusion:**

The flexibility of the ATS workflow, which enables reoptimization of the treatment plan, also introduces potential critical points where errors can occur. There are more opportunities for error in ATS that can create unintentionally negative dosimetric impact. FMEA can help mitigate these risks by identifying and addressing potential failure points in the ATS process.

## 1 INTRODUCTION

Adaptive radiotherapy offers a number of benefits, most notably permitting adjustments of the radiation plan to account for daily anatomical changes of a tumor and surrounding tissues.^1^, ^2^, ^3^ The Elekta Unity MR‐Linac (MRL) system provides adaptive functionality through an onboard 1.5 T MRI scanner which offers superior soft tissue contrast compared to conventional X‐ray guidance and therefore allows daily target and normal tissue modifications.^4^ Two forms of online adaptations are available on the MR‐Linac: adapt‐to‐position (ATP) and adapt‐to‐shape (ATS).^5^

ATP adapts the plan solely based on the patient's position on the treatment table, shifting the field apertures and weights of an earlier reference plan to match the daily target position (also referred as virtual couch shift) to treat patients with minimal interfractional changes, rather than adapting to anatomical changes of the day.^5^ Because there is no recontouring, the major failure modes for ATP arise primarily from discrepancies between the anatomy in reference conditions (i.e., simulation) and the anatomy of the day. Items that scored high in the Failure Modes and Effects Analysis (FMEA) of the ATP workflow included bringing in the wrong patient, transferring the wrong scan, improper fusion, or evaluation of fusion, and viewing the wrong image.^6^

ATS can account for larger changes and internal organ motion by allowing the user to fully recontour and reoptimize a plan.^5^ It is also a more labor‐intensive process than ATP, with appointment typically between 60 and 90 min (as opposed to about 30–45 min for ATP).

At our clinic, the ATS workflow consists of two potential pathways. The standard Elekta online workflow as described in this report refers to the use of “online Monaco” for fusion, contour transfer/editing and planning. Our clinic also employs a “MIM workflow” using MIM Maestro (MIM Software, Cleveland, OH) for fusion and contouring.^7^ In this workflow, fusion and contouring are done in MIM, then sent to an offline Monaco treatment planning system for adaptive planning. The offline version of Monaco is used in this step because the online version is behind the Elekta firewall and not on the external network thus, unable to accept DICOM‐RT structures from MIM. The MIM workflow converges with the online Monaco workflow during the verification step. The advantages of the MIM workflow is that it allows for integration of multiple MRI sequences for optimal contouring,^8^, ^9^ in‐house AI auto‐segmentation pipeline^10^, ^11^ to expedite contouring, and easy hand‐offs between planners and physicians while contouring remotely in a local workspace.

In certain situations, it may be necessary to perform a second adaptation during the same adaptive fraction and, in such cases, users may choose a third option for adaptation called ATS‐lite^12^ in place of ATP or ATS. “ATS‐lite” may be preferable to ATP in the case of large shifts and/or to account for greater motion. ATS‐lite technically uses the ATS option in online Monaco, but only includes adjustments of the body contour (with all other contours propagated rigidly). Gupta et al. has shown that ATS‐lite is a more robust adaptation method than ATP for motion ≥ 5 mm but bypasses the need for a full ATS.^12^

Efficiency in online adaptive treatment is crucial, as planning is performed while a patient is on the table (i.e., “online”). However, a complex workflow such as ATS requires active input from a multi‐disciplinary team which, combined with short timeframes, creates potentially stressful scenarios that are prone to errors. Risk analysis is critical in this context to identify potential failure modes and implement mitigations to prevent them from occurring. However, there are currently no risk analysis studies for the ATS workflow that consider both standard and MIM workflows.

This report describes a risk analysis of the Elekta Unity ATS workflow as used at our institution. The risk analysis process was based on the guidelines provided by AAPM Task Group 100 (TG‐100).^13^ Both the standard Monaco only pathway and a pathway that uses MIM are included in the analysis. The goal of this study seeks to address the current lack of FMEA studies for the ATS workflow and to minimize potential areas for failures or errors during treatment.

## 2 METHODS/MATERIALS

Members of the MRL treatment team participated in this risk analysis including one therapist, two physicians, three dosimetrists, and eight physicists. This risk analysis team developed the ATS process map, reviewed potential failure modes for each step in the process, and planned for how to mitigate failure modes. Risk analysis for this workflow was completed with Monaco version 5.51 (Elekta AB, Stockholm, Sweden), MOSAIQ version 2.83 (Elekta AB, Stockholm, Sweden) and MIM Maestro version 7.1. This analysis was done prior to the implementation of Elekta Comprehensive Motion Management (CMM) so no automated motion management was available for treatment.

For each step in the process map, the risk analysis team leveraged their clinical experience to determine potential failure modes prospectively and retrospectively based on prior near miss events. After compiling a list of failure modes, all members independently scored each item assuming no quality assurance procedures in place, using an integer score from 1 through 5 for likelihood of occurrence (O), severity (S), and detectability (D). This adjustment from the 1 to 10 scale used in the task group report was made to help simplify scoring. The product of O, S, and D creates a risk priority number (RPN) which is used to identify risks to the patient. The failure modes were then ranked in four different categories: average RPN, average S, median RPN and median S. The top 20^th^ percentile of failure modes in each category (“high‐ranking”) were isolated for mitigations.^13^ The team developed mitigations by evaluating current safety procedures for their efficacy at mitigating failure modes, adjusting current workflow procedures, and adding additional prevention methods for failure modes if necessary. Suggested mitigations were then reviewed independently by the group for a final round of comments.

To simplify the analysis of failure modes that could have varying degrees of severity, our team categorized failure modes as “major issue” or “minor issue.” A “major issue” is one that would have a significant dosimetric impact whereas a “minor issue” would not. For example, incorrectly contouring an organ that is far from the treatment region would be a minor issue, while incorrectly contouring a dose limiting organ would be a major issue.

**FIGURE 1:**
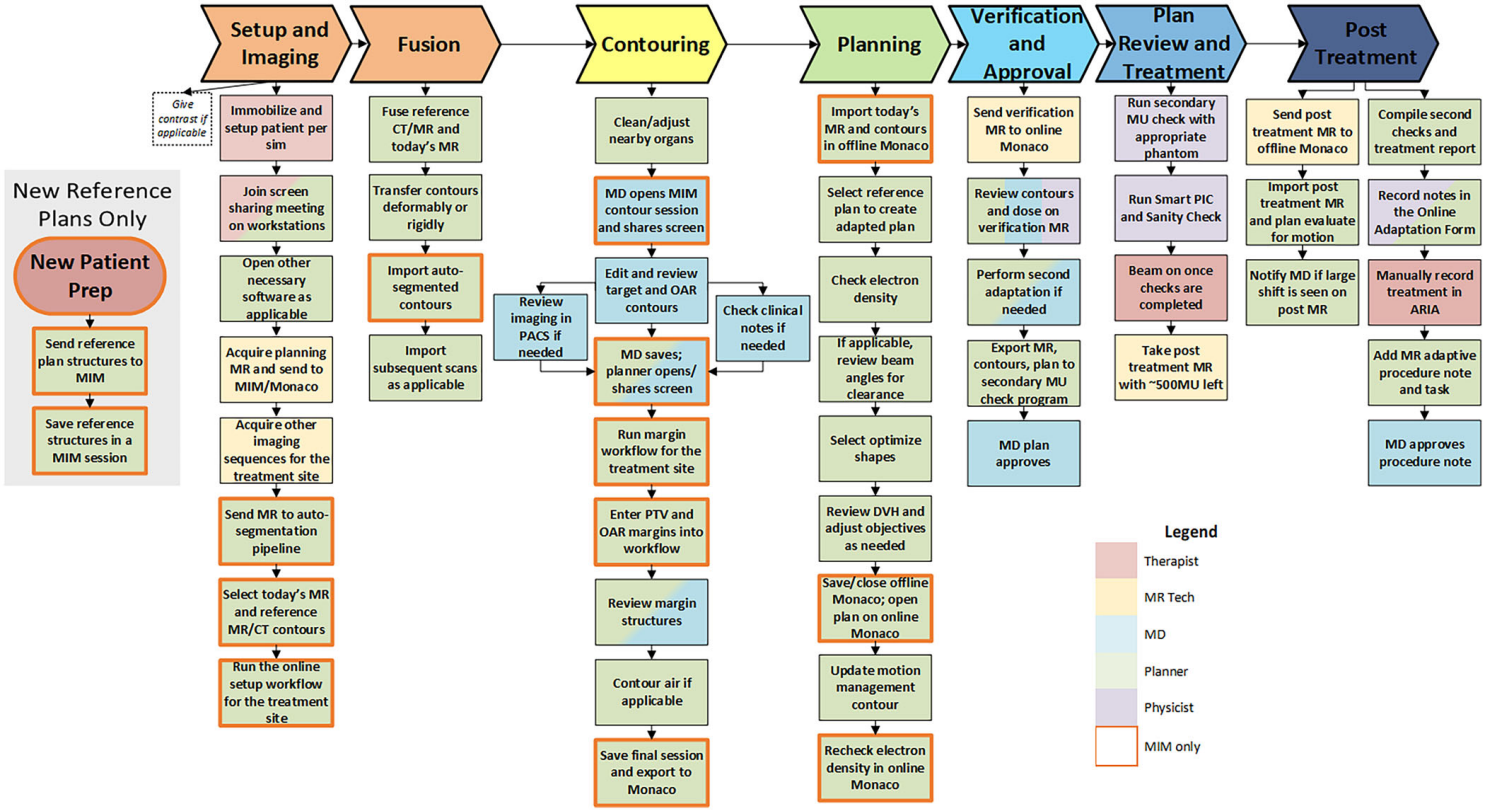
ATS Process Map; each task is indicated by a block along the process map and colored based on who typically performs this task. MIM workflow tasks are highlighted with a bold edge.

The process map for ATS treatments begins after the reference plan review and covers all the steps to create a fully adapted plan. During a single adaptive fraction, users may need to switch between the MIM workflow and the online Monaco workflow due to specific clinical needs, as it may be more efficient later in the process to rescan and make contour edits in online Monaco rather than returning to MIM. Consequently, the process map treats both pathways in parallel and categorizes the ATS workflow into seven steps summarized in Figure 1.

## 3 RESULTS

**FIGURE 2:**
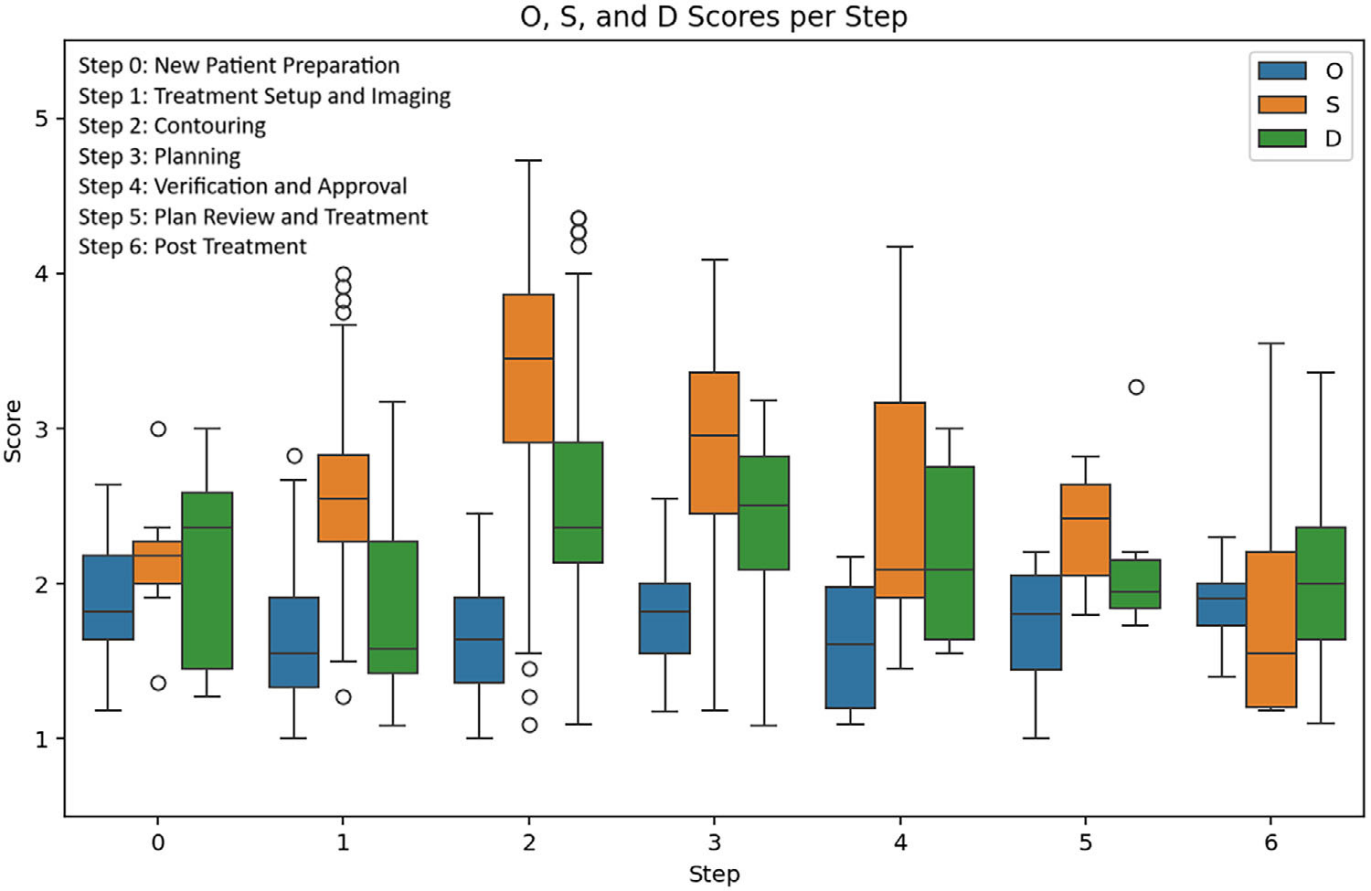
Averaged O, S, and D scores for all 264 failure modes for each step in the process.

A total of 264 failure modes were identified, and mitigations were discussed for 82 of them. High ranking failure modes were found in each step of the ATS workflow as shown in Table 1. The averaged scores for all failure modes per step can be seen in Figure 2. High‐ranking failure modes for each step are summarized in the following sections. A comprehensive table of failure modes with scores can be found in Supplemental Materials.

**TABLE 1:** ATS FMEA results showing the number of sub‐steps, failure modes, and high‐ranking failure modes for each step.

Step number	Step description	Number of sub‐steps in step	Total failure modes	High‐ranking failure modes
0	(MIM only) Export reference plan structures to MIM	2	7	1
1	Position patient and take and fuse image of the day with the reference image	15	89	10
2	Recontouring of structures based on image of the day	17	75	46
3	Reoptimization of the treatment plan based on structures of the day	12	42	16
4	Take and review a verification MR to check for motion and plan approval	6	22	6
5	Physicist plan review and patient treatment	3	10	1
6	Post treatment tasks	11	19	2

### 3.1 Step 0: New patient preparation

The highest‐ranking failure mode for this step is failure to send the most updated structure set from Monaco to MIM. For each initial reference plan, the final version planning contours used in Monaco are sent to MIM as a starting point for adaptation. Because the adapted plan depends on matching structure names to transfer average electron density (ED) information, dose‐volume histogram (DVH) statistics, and planning objectives, mismatched structures could result in missed DVH constraints or optimization objectives. This failure mode was in the 20^th^ percentile for both highest average RPN score and median severity.

### 3.2 Step 1: Treatment setup and imaging

Most of the top‐ranking failure modes in this step involved improper placement or usage of the compression belt, failing to limit respiratory motion. Without an automated motion management system at the time of risk analysis, we require tumor motion to be <0.5 cm to be eligible for treatment on the MRL. When tumor motion is determined to be >0.5 cm at simulation, a compression belt is used to limit tumor motion for abdominal treatment sites.^14^, ^15^ While the use of the compression belt may be reduced with the availability of gating, it may still be used in certain situations and thus failure modes are summarized in Table 2.

**TABLE 2:** Compression belt failure modes and mitigations.

Failure mode	Mitigation
Patient's breathing pattern does not match simulation:	Primary mitigation is proper motion assessment at simulation.
	One may slightly increase belt pressure during first fraction if patient can tolerate.
Belt placement incorrect:	Marks on patient mold to indicate placement of the belt. Placement cannot be standardized due to uniqueness of patient anatomy and needs.
	Imaging would reveal placement or pressure mismatch issues.
Inconsistent belt pressure due to leaks or improper use:	Therapists and MR technicians receive training for proper belt use.
	Pressure gauge constantly watched by therapist during treatment.
Wrong pressure or no pressure used:	Constant monitoring of pressure gauge would catch this failure mode
Belt missing or forgotten:	Compression belt use is standard for all abdomen patients in our clinic.^a^
	Imaging would reveal no compression belt was used.
Belt not documented in setup notes:	Two simulation therapists independently verify the accuracy of setup document.

^a^ Clinic standards for abdomen may change in the future with the availability of gating.

It is also possible that even with proper compression belt use, tumor motion is greater than tolerance due to incorrect motion assessment during simulation. Improper motion assessment is another high‐ranking failure mode. An example of how tumor motion is assessed may be found in Supplemental Materials.

Finally, to allow for the use of either MIM or offline Monaco, automatic image importing from the scanner is disabled in our clinic. As images must be manually exported and imported, using an incorrect image for adaptive planning is another high‐ranking failure mode.

### 3.3 Step 2: Contouring

Many of the high‐ranking failure modes relate to contour accuracy. A failure mode that scored high in average RPN, average severity, and median severity was that contours were adjusted using the wrong scan. This could mean either an inappropriate sequence was used to identify a structure or a scan from a different timepoint was used, both of which result in contours that do not accurately represent the current anatomy. The magnitude of the error could increase if the scan used for contouring was from a different day or if the suboptimal MR sequence causes over or under contouring of tissue near the treatment region. It is also possible to misidentify an organ or tumor due to inexperience with MR images or simply human error (e.g., selecting the wrong contour to edit). Additionally, when not properly contoured, air (e.g., bowel gas) poses specific dosimetric concerns.^16^

Failure modes regarding incorrect contour margins also scored in the top 20^th^ percentile for highest average RPN, median RPN, average severity and median severity. Certain structures are created using a semi‐automated expansion and/or subtraction process. In the online Monaco only workflow, the “recreate margins” tool is used to add, expand, contract, or crop structures with a predefined formula. To do this, the structure must be designated as a “margin” structure; errors are introduced if the wrong contour type is selected. This failure mode could occur if the wrong reference plan is selected, if errors existed in the reference plan, or if planners forget to use the recreate margins tool. In the MIM workflow, PTV and PRVs are generated using a custom site‐specific workflow, but expansion or subtraction margins must be manually entered to allow for clinical flexibility (an example of this workflow is shown in Supplementary Materials).

### 3.4 Step 3: Planning

High‐ranking failure modes in this step include choosing the wrong reference plan for adaptation, structure name mismatches, incorrect EDs, and DVH template issues. An incorrect reference plan has cascading effects for subsequent steps as it contains the EDs, optimization objectives, and the DVH template used to evaluate target goals and organ constraints. If the wrong reference plan is selected, any changes to ED assignment, optimization objectives or DVH goals will not be reflected in the adaptive plan. Similarly, if Monaco is unable to match a contour with that of the selected reference plan, the structure will not be given an ED assignment, optimization objective or DVH goal. Additionally, ED assignment also depends on the structure layering list, with the top layer “owning” the voxels within its contour, which leads to an additional failure mode if the layering is incorrect. An example from our clinic (which was discovered and corrected in initial chart check) was the incorrect layering of a large structure over the external that resulted in an incorrect average ED assigned to areas that were not contoured (e.g., fat, muscles). DVH template issues typically stem from structure mismatch issues or changes in the reference plan. Having either incorrect or missing optimization objectives and DVH constraints may result in a plan that does not meet treatment goals.

Another high‐ranking failure mode is forgetting to update the motion monitoring (MM) structure before plan approval (with implementation of CMM, other motion monitoring functionalities are available which are not addressed here). The MM structure highlights the area of interest for monitoring during treatment by cine MRI and so needs to be accurate such that the treatment can be paused if necessary. Since ATS allows for contour editing, the MM structure may be changed to account for interfractional changes. However, once the structure is changed, the user must click on the “update MM” button to invoke the changes on the cine MRI, and forgetting to do this is a high‐ranking failure mode. If the MIM workflow is used, the MM structure is updated as part of the margin workflow.

### 3.5 Step 4: Verification and approval

Before plan approval, verification imaging is acquired and assessed to evaluate whether motion has occurred since initial scans and if readaptation may be necessary. Reviewing the wrong image during verification image analysis scored as a high‐ranking failure mode.

Another high‐ranking failure mode that ranked in the top 20^th^ percentile in all categories is, due to inadequate review of the verification image, ATP was used when ATS or “ATS‐lite” would have been more appropriate because of excessive anatomical discrepancies. This can lead to a suboptimal plan as ATP cannot adequately account for large displacements or internal deformation.

### 3.6 Step 5: Plan review and treatment

A high‐ranking failure mode for this step is that cine MRI, used for motion detection during treatment, is stopped prematurely to acquire a post treatment scan, thus allowing a significant amount of dose to be delivered with potentially unacceptable motion that will not be detected until this scan is complete. In the present workflow (which was defined prior to release of CMM), a MM structure is used to visually monitor an area of interest, such as the tumor. However, this monitoring only includes three planar views at the center of the MM structure with no ability to change the image plane. For certain cases, it may be necessary to review a 3D image to assess 3D motion and review dose to organs/tumor according to intra‐treatment scans. To allow for this, cine MRI is turned off and a post treatment scan is acquired while treatment is completed (generally started with approximately 500 MU remaining).

### 3.7 Step 6: Post treatment

A high‐ranking failure mode for this step is failure to notice significant motion due to inadequate review of the post treatment scan. Review of the post treatment MRI helps to identify if a large motion has occurred which may require correction or further monitoring for the following fraction. With prostate treatments, since the MM is typically the tumor, it is easy to catch if the patient moved or if bladder or rectal filling displaced the organ. However, for treatment sites such as pancreas, both the tumor and organs can move internally without external patient movement. In complex cases, there is a risk of overlooking the evaluation of a structure, potentially resulting in the inadvertent inclusion of a dose‐limiting organ within the treatment region.

## 4 DISCUSSION

Mitigations for the high‐ranking failure modes are summarized in this section. Compression belt mitigations are summarized in Table 2. A full list of all the high‐ranking failure modes and their mitigations are found in Table 3.

**TABLE 3:** High ranking failure modes and mitigations from the adapt‐to‐shape workflow risk analysis.

Step	Process map step name	Potential failure mode (s)	Potential causes of failure	Mitigation(s)
**0**	After reference plan approval, send final structure set to MIM	Changes made to structures in Monaco after sending	Did not send the most updated version	(1) finalization workflow specific to anatomic site to compare structure names from margin workflow contours vs. Monaco export; (2) reference plan structures sent after plan approval; (3) target changes/additions discouraged without replanning
**1**	Place immobilization devices and setup patient per simulation notes	Belt placed correctly, but residual motion > 0.5 cm even with belt placed	Sim did not measure breathing excursion in the right place	(1) by default, measure at ISO location so remeasurement can be done in similar location; (2) train therapists on where to measure motion or find a good surrogate for motion at sim; (3) motion is verified with MD; if motion is high, physics is also contacted
			Patient breathing differently than sim with the belt on	Pressure can be slightly adjusted at 1st fraction, if it is tolerable by the patient, but primary mitigation is to have proper assessment at the time of simulation
		Setup position level does not match sim	Unintentionally placed patient in wrong position	(1) reference marks are placed on mold during sim; (2) belt visible on MRI scan, can compared to planning scan
		Placed compression belt but no air so breathing is not restricted	Leak in system	(1) MRI techs and therapists are trained in the use of the belt; (2) pressure gauge is constantly watched during treatment
			Pumped to wrong pressure	(1) pressure is initially verified against setup notes; (2) body differences in images can be compared to planning scan
			Forgot to pump air	(1) MRI techs and therapists constantly watch pressure gauge; (2) belt visible on MRI scan when compared to planning scan
		Did not put compression belt	Not in setup notes	(1) sim setup for GI MRL is standardized; (2) two therapists verify documentation is accurate
			Forgot to put on	(1) GI sim standard for MRL has compression belt; (2) will notice belt missing on MR scan
			Did not know a belt is used	Therapist training include learning about simulations standards and competency tests
	Import subsequent scans as applicable to Monaco	Imported scan from the wrong day (Monaco)	Selected the wrong scan date to bring into the current session	(1) online Monaco won't auto‐populate image scan older than current scan; (2)call‐out procedure to verify sequence, patient, date, and time
**2**	Planner to adjust OARs and CTV as applicable	Major Issue: Adjusted incorrectly	Wrong scan used to adjust contours	(1) MD contours organs close to target; (2) independent review of contours by physicist and planner; (3) GI MDs actively draw critical OARs so will know if the sequence is best for OAR
			Misidentified organ	(1) independent review of contours by physicist and planner; (2) planner does not adjust targets but MD shares screen during contouring
		Major Issue: Did not adjust	Forgot to adjust OAR	Independent review of contours by physicist and planner
	MD shares screen and opens MIM session	Opened wrong session	Mistakenly clicked on the wrong session	(1) confirm scan date of opened session (2) lock all old MIM sessions when sending final session to Monaco (3) plan checker verifies sessions are locked when performing independent review of contours in MIM
			Instructions unclear/had the wrong instructions	(1) session locking procedure gives warning if opening session from another day (2) if MD opens older session from same day, desired images are missing
	MD reviews CTV, GTV, and OARs and edits as needed	Major Issue: Edits the wrong structure (i.e., wrong structure selected)	Edited incorrect OAR (e.g., drew large bowel on small bowel)	(1) planner to watch MD while they are contouring and to inform MD if wrong structure is selected; (2) physicist and planner independently review contours so will catch incorrect naming
		Major Issue: Edited structure incorrectly	Misidentified target	Target contours are copied to the new image rigidly and can be used as reference
			Misidentified organ	(1) physicist and planner independently review contours; (2) for GI, patients drink water before imaging to provide contrast between duodenum and pancreas head
			Wrong scan used to adjust contours	(1) physicist and planner independently review contours; (2) have policy to always double check active image in view
		Major Issue: Did not edit structure that required adjustment	Forgot to edit target contour	Planners record tumor volume after each fraction for all sites, so will catch after treatment, that the volume did not change
			Forgot to edit OAR contour	(1) physicist and planner independently review contours so will catch contour mismatch with image; (2) the safety culture encourages all to ask if something looks wrong
		Major Issue: Edits the wrong structure (i.e., wrong structure selected)	Edited incorrect OAR (e.g., drew large bowel on small bowel)	
			Edited OAR but target selected (e.g., drew small bowel on GTV contour)	Physicist and planner independently review contours so will catch incorrect naming
			Edited target but OAR selected (e.g., drew GTV on small bowel contour)	
		Minor Issue: Edits the wrong structure (i.e., wrong structure selected)	Edited target but OAR selected (e.g., drew GTV on vessels)	(1) physicist and planner independently review contours so will catch contour mismatch with image; (2) the safety culture encourages all to ask if something looks wrong
			Edited OAR but target selected (e.g., drew vessels on GTV)	
		Minor Issue: Edited structure incorrectly	Misidentified organ	
			Misidentified target	Target contours are copied to the new image rigidly and can be used as reference
			Wrong scan used to adjust contours	(1) physicist and planner independently review contours; (2) always double check active image in view
		Minor Issue: Did not edit structure that required adjustment	Forgot to edit OAR contour	
			Forgot to edit target contour	Tumor volume is recorded after each fraction for all sites, so will see that the volume did not change
	MD to check clinical notes if needed (e.g., covering for another MD or does not remember patient)	Did not check and it was needed	Forgot	MD have access to PACS, EMR, MIM during online workflow so they could look during the adaptation process if needed but it will increase time on the table for the patient
		Checked but looked at the wrong reference material	Did not look at newest information	
			Mistakenly looked at the wrong information	
	MD to check imaging in PACS if needed	Did not check and it was needed	Forgot	
		Checked but looked at the wrong reference material	Did not look at newest information	
			Mistakenly looked at the wrong information	
	Planner opens MD saved session and shares screen	Opened wrong session	Mistakenly selected the wrong session	After saving “for MD” session, note the number of sessions at the top and then always open the most recent session (saved by MD) and check that the number of sessions updated
			Instructions unclear/had the wrong instructions	
	Enter PTV margins	Inputted wrong values	Mistakenly typed wrong number for target margin	(1) contours are reviewed with MD; (2) physicists also independently check contour; (3) physicist to measure margins with ruler during independent review
			Wrong PTV cropping done	
			Instructions unclear/had the wrong instructions	(1) plan checker reviews margins between checklist and contours, measuring margins with ruler
	Enter OAR margins if applicable	Inputted wrong values	Mistakenly type wrong number for OAR cropping	Physicist to measure margins with ruler during independent review and compare with online checklist
			Mistakenly typed wrong number for OAR margin	
			Instructions unclear/had the wrong instructions	
	Review margin structures with MD	Reviewed but did not catch error	Did not review thoroughly	(1) physicist reviews contours independently; (2) planner should call‐out margin used for PTV when reviewing contours; (3) box on checklist to verify this was done
		For online Monaco only workflow: wrong generation method for contour not discovered (i.e., rigid instead of margin type)	Reference plan incorrect	(1) contours are reviewed with MD; (2) physicists also independently check contour
		Margin structure is blank	Did not click “recreate margins”	
	Contour air if applicable	Did not contour but structure is needed	Images were too old/not updated to reflect air bubble in treatment area	(1) only contour on most recent air scan; (2) for prostate, will see air on MM since have it on before beam is on
			Images did not show that air bubble was in the treatment area (non‐optimal sequence to show air)	Have standardized sequence called “air scan” and is the only one available
		Did not use air scan to contour air	Thought was looking at air scan but wasn't	(1) air scan image is very different than typical scans; (2) in MIM, sequence name is displayed
	Export today's MRI and contours to offline Monaco	Exported wrong MR/contours	Mistakenly selected the session from the wrong date	(1) for older images, Monaco will import but under older study ID; (2) planner checks date in import window; (3) physicists to check image properties when doing independent review
			Mistakenly selected the “For MD” session from today	(1) Monaco will not import structure set if not match with image; (2) for images from the same date but an older session, PTVs will be missing; (3) review image details to confirm date
			Mistakenly selected the “MD approved” session from today	Structures from previous sessions won't have PTV so will be a hard stop in Monaco
	In offline Monaco, import today's MRI and contours	Import wrong scan/structures	Wrong scan exported	(1) for older images, Monaco will import but under older study ID; (2) planner checks date in import window; (3) physicists to check image properties when doing independent review
			Old scan present in folder	
**3**	Select reference plan to create adapted plan	Choose wrong reference plan	Mistakenly selected wrong plan	Planner calls out MRI number and plan name number when creating adapted plan
	Check ED assignment on adapted plan	ED assignment incorrect	Structure name inconsistent with reference plan so not recognized in adapted plan	(1) check contour names against reference plan report; (2) have online checklist that gives note about changes needed; (3) have “mismatch in structure name” error for DVH and optimization; (4) ED will be body and physicist does independent ED check
			Layer of structures incorrect (not external)	(1) plan checkers review structure layers; (2) physicist independent review of ED for air, bone, spinal canal, lung, and tissue by color wash and point samples
			Structure requiring override does not have correct ED value not caught (e.g., air not assigned or missing)	
			Large structure over external making average ED incorrect where there are no contours (e.g., fat, muscles)	(1) physicist reviews structure layers initial plan checking; (2) body contour should always be at the bottom of the list
		Did not check ED assignment	Forgot	(1) physicist checklist reminder to perform ED check; (2) check on average ED for all structures (not lung, air) with individual check for lung and air—requires separate script
	Check beam angles/arrangement	Arms in the way of beam	Setup does not match simulation	(1) arm position is checked during fusion; (2) turn on fields when viewing ED to check all together
	Check DVH and adjust constraints while optimizing	Major Issue: Optimization objectives incorrect	Changed in reference plan and not noticed	(1) compare DVH template with reference plan during optimization; (2) constraint changes noted in online checklist after each fraction
		Major Issue: Mismatch of structure name causes missing objectives (e.g., PTV or nearby OAR)	Changes in Monaco after export to MIM	Monaco gives structure mismatch error and inability to optimize
			Changes in MIM causing mismatch with Monaco	
	Check DVH and adjust constraints while optimizing	Major Issue: DVH template has incorrect constraints	Changed in reference plan and not noticed	(1) compare DVH template with reference plan during optimization; (2) constraint changes noted in online checklist after each fraction
		Minor Issue: DVH template has incorrect constraints		
	Check DVH and adjust constraints while optimizing	Major Issue: Mismatch of structure name causes missing DVH criteria	Changes in MIM causing mismatch with Monaco	Monaco gives structure mismatch error and inability to optimize
			Changes in Monaco after export to MIM	
	Update MM	Did not update MM	Forgot to update MM	(1) in MIM workflow, MM Is updated automatically; (2) physicist does independent review of contours
	Recheck ED in online Monaco	Did not recheck ED	Forgot to recheck but checked in offline Monaco	ED thoroughly checked during offline Monaco process
**4**	Send verification MRI to online Monaco	Wrong images sent to Monaco	Wrong sequence from today exported from MRI scanner	MRI Techs do “call‐out” procedure of date, sequence, time and destination
			Wrong image date exported from MRI scanner	
	Scroll and review contours and dose on verification MRI with MD	Wrong image used for review	Looked at secondary image instead of primary	Double check “show images” window
		Missed large patient shift	Did not redo ATS when it was needed	Physicists and planners and MD independently review verification scans
	If needed, perform second adaption	Organ/tumor significantly changed such that it is not accurately represented by contour and did ATP instead of 2nd ATS (i.e., bowel moved)	Missed in review of verification scan	(1) verification scan is standardized as part of the workflow; (2) verification scan in physics checklist; (3) if there is large motion, will see it on the MM (have designated MM watcher)
		ATP performed when ATS‐lite should have been used due to magnitude of shift	Did not know to use “ATS‐lite”	(1) have policy to use ATS‐lite if shift > 5 mm; (2) planner/physicist trained that ATS‐lite is only for external and anything else needs to notify MD
**5**	When ∼500MU is left, take post treatment MRI if needed	Started post treatment scan too early, patient moves so it is not caught on MM	Unaware of policy	(1) training of therapists includes required sequences for each site; (2) requirement for post MRI standard between sites
**6**	In Offline Monaco: review contours from treated plan on post MRI	Incomplete review of the post MRI	Reviewed and thought image looked ok but there was a large change/shift not noticed by the planner	(1) review verification scan with physicist & MD so organ motion concerns only during beam on; (2) if target if MM structure (e.g., prostate), can catch motion during treatment; (3) checklist for post MRI review to check critical organs and notify MD for large shifts
	Notify MD if large shift has occurred	Did not notify MD	Forgot	

*Note*: Some failure modes may have multiple mitigations and are numbered. Numbers only serve to separate ideas and are not an indicator of value.

Abbreviations: CTV, clinical target volume; DVH, dose volume histogram; EMR, electronic medical record; GI, gastrointestinal; GTV, gross tumor volume; ISO, isocenter; OAR, organ at risk; PACS, picture archiving and communication system; PTV, planning target volume.

While ATS begins with the patient's treatment setup, some mitigations need to be implemented prior to this step. At simulation, proper tumor motion evaluation is crucial to ensure patient eligibility for MRL treatment. A finalization workflow in MIM (an example is found in Supplemental Materials), executed after the initial reference plan is complete, ensures that the most updated structure set is used for adaptation and helps to prevent structure mismatch issues that may occur later in the processes. A department wide standardization of contour names, such as the implementation of TG263^17^ can also help to mitigate structure mismatch issues. Thorough review of assigned EDs during both reference plan creation and the physicists initial chart review can catch layer ordering failures or incorrect ED assignments. The body/external contour should always be at the bottom of the layer list with an average ED close to 1. Comprehensive training on the MRL workflows helps to mitigate treatment day issues by ensuring staff are familiar with the typical MR sequences, their timing within the process, and how to respond to various readaptation conditions. Moreover, a well‐established culture of safety is also crucial at preventing failures in the workflow. All staff involved in the patient's treatment should be encouraged to ask questions and report concerns if they believe something was done incorrectly, even if it falls outside their usual scope of work. For example, if a planner perceives that a physician has used a suboptimal MR sequence to contour an organ, they should feel empowered to raise the issue during the contouring.

The call‐out procedure^6^ is useful for mitigating high‐ranking failure modes associated with incorrect reference plan and image set selections. When sending images from the scanner to either Monaco or MIM, the patient's name, sequence name, scan date, scan time, and destination should be called out by the MR technician and verbally verified by others in the room. Calling out the image time is important to ensure the most recent image is sent for contouring editing and verification image review, as it accurately reflects internal organ positioning. Similarly, the planner should “call‐out” the reference plan number and MRI number associated with the chosen plan, as well as the manually entered numerical values used for margin structures.

Along multiple points in the ATS planning process, independent checks done by different team members can help to mitigate many of the high‐ranking failure modes. A specialized 3D air sequence is acquired prior to finalizing the contours^18^ and is reviewed by the MRI technician/therapists and physicist. Should a large amount of air be present, it must be removed, reduced, or accounted for in ED assignments. Prior to the physician's final approval of the structures, the physician, planner, and physicist thoroughly review each contour for accuracy, including the name of the contour and the date and sequence of the image in view. Special attention should be given to margin structures, C‐shaped and donut structures for contour accuracy. If the MIM workflow is followed, the physicist opens the final MIM session in parallel while the planner continues to optimization. If only online Monaco is used, this review is done prior to moving on to optimization. To check for internal motion immediately before treatment, the verification image should also be independently reviewed by the physicist, planner, and physician in a thorough manner with contours turned on and attention given to the image time to ensure that the correct image is in view.

Checklists, when following AAPM MPPG4a^19^ guidelines, are used as reminders for some high‐ranking failure modes and to communicate information for subsequent fractions. A checklist is used by the physicist as reminders for independent contour and imaging reviews, ED accuracy checks for air, bone, tissue, and spinal canal, and to update the MM structure. A suggested mitigation is to add a separate checklist for post treatment MRI review to remind the planner to review critical organs such as small bowel, large bowel, and stomach and to notify the physician should the planner notice a large motion. The patient specific online checklist^6^ include updates to structure names, optimization objectives or DVH template goals to ensure the correct reference plan is selected and prevent structure mismatch errors. The online checklist also records GTV or CTV volumes to reduce the risk of forgetting to edit the target or unintentionally creating inappropriate edits to the target. For each fraction, target volumes can be compared for unexpectedly large changes or, if there was no change, a potential indicator that the contour was unintentionally not adjusted. The online checklist also serves as a platform for special reminders such as those for non‐standard post treatment imaging.

## 5 CONCLUSION

Standardization minimizes interfractional and inter‐patient variations, reducing the likelihood of errors. Additionally, implementing duplicate checks, particularly those conducted independently by another team member, helps identify and correct errors early, preventing them from progressing further in the workflow. An efficient workflow with a data driven set of mitigations is essential for safe and effective treatment. An FMEA analysis was essential to identify the most crucial steps and ensure mitigations are in place to significantly reduce the likelihood of failures.

## AUTHOR CONTRIBUTIONS

All authors listed actively participated in the risk analysis process for the Adapt‐to‐Shape workflow on the Unity. This included reviewing the process map, failure modes, causes of those failure modes, independently scoring each of the high‐ranking failure modes and finding mitigations for those failure modes.

## CONFLICT OF INTEREST STATEMENT

The authors declare no conflicts of interest.

## Supporting information



Supporting Information

Supporting Information

Supporting Information
