# Supplementary material for: Risk analysis of the Unity 1.5T MR‐Linac adapt‐to‐shape workflow
Source: J Appl Clin Med Phys. 2025 Apr 16;26(7):e70095. doi: 10.1002/acm2.70095 (PMC12256694; doi:10.1002/acm2.70095)
Supplement: Supplementary file 1 — Supporting Information [file ACM2-26-e70095-s001.pdf]

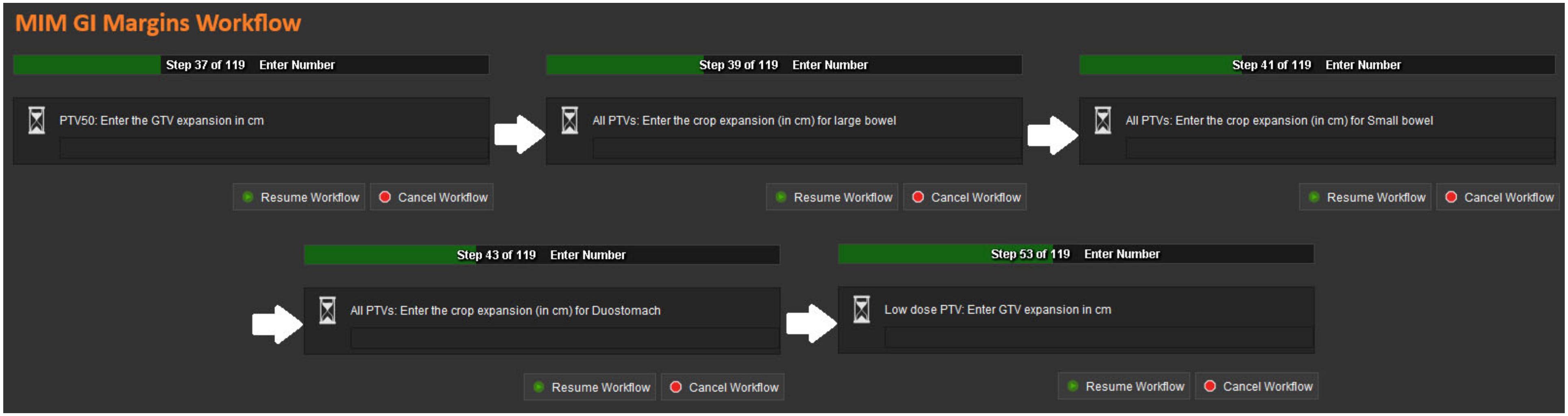

Example of the workflow in MIM for recreating margin structures in gastrointestinal (GI) treatment sites. Each pop-up window prompts the user to enter a value to use either for the expansion a crop distance for PTV to an organ. In this example, the treatment includes a simultaneously integrated boost (two dose levels). The user first enters a GTV to PTV margin for the higher dose PTV, then enters distances to crop the higher dose PTV from large bowel, small bowel and stomach. Finally, the user enters a GTV to PTV margin for a lower dose PTV.
