# Supplementary material for: Risk analysis of the Unity 1.5T MR‐Linac adapt‐to‐shape workflow
Source: J Appl Clin Med Phys. 2025 Apr 16;26(7):e70095. doi: 10.1002/acm2.70095 (PMC12256694; doi:10.1002/acm2.70095)
Supplement: Supplementary file 2 — Supporting Information [file ACM2-26-e70095-s002.pdf]

[ATS MIM and Online Monaco Workflow on MR-Linac]

MIM workflow steps have green background

Step Num.	Process Map Step Name	Potential Failure Mode(s)	Potential Causes of Failure	O avg	O med	S avg	S med	D avg	D med	RPN avg	RPN med
0	**New Plan Preparation**										
	After reference plan approval, send final structure (reference plan) set to MIM	Did not send	Forgot	1.73	2.00	2.18	2.00	1.27	1.00	4.50	3.00
		Changes made to structures in Monaco after sending	Did not send the most updated version	2.09	2.00	2.36	2.00	3.00	3.00	15.75	8.00
		Sent wrong structure set	Planned on wrong structure set	1.18	1.00	3.00	3.00	2.73	3.00	8.33	6.00
		Couch is not removed and sent to Monaco as a double couch structure	Planner forgot	1.55	2.00	1.91	2.00	1.64	1.00	4.08	4.00
		Structures not named properly for online workflow	Planner Forgot	2.64	3.00	2.18	2.00	2.36	2.00	14.00	7.00
			Renamed but used the wrong name	2.27	2.00	2.09	2.00	2.45	2.00	13.33	7.00
	Save structure set with easily recognizable name	Did not rename	Forgot	1.82	2.00	1.36	1.00	1.27	1.00	3.92	2.00
1	**Setup & Imaging**										
	Place immobilization devices and setup patient per simulation notes	Did not put compression belt	Forgot to put on	1.17	1.00	3.83	4.00	1.42	1.00	5.67	5.00
			Not trained to know a belt is used	1.17	1.00	3.75	4.00	1.58	1.00	6.42	5.00
			Not in setup notes	1.33	1.00	3.92	4.00	1.92	1.50	9.42	7.00
		Placed compression belt but no air so breathing is not restricted	Forgot to pump air	1.33	1.00	3.67	3.50	1.58	1.50	7.25	6.00
			Pumped to wrong pressure	2.17	2.00	3.33	3.00	2.42	2.50	17.08	18.00
			Leak in system	1.58	1.00	3.58	3.50	2.83	3.00	16.58	13.50
		No patient mold	Forgot to put on	1.00	1.00	3.42	3.00	1.58	1.00	4.92	5.00
			Not in setup notes	1.08	1.00	3.42	3.00	1.92	1.50	6.50	5.00
		Setup position level not match sim	Unintentionally placed patient in wrong position	2.17	2.00	3.00	3.00	2.00	2.00	15.83	10.50
			Wrong index in setup notes	1.83	2.00	3.00	3.00	2.25	2.00	12.83	8.50
		Belt placed correctly, but residual motion > 0.5cm even with belt placed	Sim did not measure breathing excursion in the right place	2.83	3.00	3.50	3.00	3.17	3.00	36.75	22.50
			Patient not breathing properly with the belt on	2.58	3.00	3.17	3.00	2.50	2.50	21.67	18.00
		Air pressure changed mid-scan	Forgot to pump air initially so have to stop scan and restart	1.58	2.00	2.92	3.00	2.00	2.00	9.08	7.00
			Therapist not trained for proper use of compression belt	1.25	1.00	3.17	3.00	2.00	2.00	7.58	7.00
	Join Webex on Sequencer and another computer (with Offline Monaco access) if applicable	MRL did not join Webex	Forgot	1.08	1.00	1.50	1.00	1.17	1.00	2.08	1.00
			Connection issue	1.92	2.00	2.08	1.00	1.33	1.00	6.25	2.50
			Appointment not on the schedule	2.67	2.50	1.67	1.00	1.17	1.00	6.58	3.00
			Forgot	2.00	2.00	2.42	1.50	1.25	1.00	5.83	4.00
			Connection issue	2.00	2.00	2.50	1.50	1.42	1.00	6.83	6.00
			Appointment not on the schedule	2.67	2.00	2.42	1.50	1.50	1.00	9.42	8.00
		Planner did not join Webex	Forgot	1.08	1.00	2.25	1.00	1.08	1.00	3.00	1.00
			Connection issue	1.50	1.00	2.33	1.00	1.17	1.00	3.92	2.00
			Appointment not on the schedule	2.08	2.00	2.25	1.00	1.33	1.00	5.33	5.00
		Physicists did not join Webex	Forgot	1.25	1.00	2.25	1.00	1.17	1.00	3.25	2.00
			Connection issue	1.58	1.00	2.42	1.50	1.25	1.00	4.33	4.00
			Appointment not on the schedule	2.08	2.00	2.33	1.50	1.42	1.00	5.67	5.00
		Joined but did not share screen	Forgot	2.08	2.00	1.58	1.00	1.08	1.00	4.50	3.00
	Open necessary software	Did not open everything needed	Forgot	1.67	2.00	1.50	1.00	1.08	1.00	3.17	2.00
	Acquire appropriate planning sequence and send to MIM/Online Monaco	Acquired a different sequence	Unintentionally selected wrong sequence	1.58	1.50	2.83	2.50	1.83	2.00	7.83	7.00
			MR protocol was changed	1.50	1.50	2.92	2.00	3.08	3.00	12.50	12.00
		Sense artifact on MR Image	Issue with coil/coil connector	1.92	2.00	3.08	3.00	2.00	1.50	12.25	8.00
			Sense image was not re-acquired between patients due to bug in MR system	2.00	2.00	2.92	2.50	2.08	2.00	12.17	11.00
		Unable to send to MIM/Online Monaco	Server issue	2.17	2.00	3.08	3.00	1.42	1.00	9.08	6.50
	Acquire and send over other sequences appropriate for the site as needed	Missing ethrive	Forgot	1.42	1.00	2.33	2.00	1.25	1.00	4.33	3.00
			Did not know which additional sequences to acquire	1.58	1.50	2.33	2.00	1.25	1.00	5.00	3.00
			Scanner Failure	1.17	1.00	2.58	2.00	1.33	1.00	4.58	3.00
		Missing IVIM (prostate)	Forgot	1.42	1.00	2.42	2.00	1.42	1.00	5.00	4.00
			Did not know which additional sequences to acquire	1.33	1.00	2.33	2.00	1.33	1.00	4.42	4.00
			Scanner Failure	1.17	1.00	2.50	2.00	1.42	1.00	4.17	3.00
		Missing Air Scan	Forgot	1.33	1.00	2.75	3.00	1.67	1.50	6.25	4.00
			Did not know which additional sequences to acquire	1.33	1.00	2.67	3.00	1.50	1.00	6.08	4.00
			Scanner Failure	1.17	1.00	2.83	3.00	1.50	1.00	5.33	4.00
		Missing urethra scan	Forgot	1.25	1.00	2.67	2.50	1.33	1.00	4.75	4.00
			Did not know which additional sequences to acquire	1.33	1.00	2.67	2.50	1.33	1.00	5.25	4.00
			Scanner Failure	1.17	1.00	2.83	2.50	1.42	1.00	5.08	4.00
		Missing MV Stir (liver)	Forgot	1.42	1.00	2.58	2.50	1.42	1.00	5.92	3.50
			Did not know which additional sequences to acquire	1.50	1.50	2.50	2.50	1.50	1.00	6.58	4.00
			Scanner Failure	1.17	1.00	2.75	2.50	1.42	1.00	4.92	3.50
		Missing fast T2 scans (GI)	Forgot	1.25	1.00	2.58	2.50	1.50	1.00	5.42	4.00
			Did not know which additional sequences to acquire	1.33	1.00	2.50	2.50	1.42	1.00	5.58	4.00
			Scanner Failure	1.17	1.00	2.83	2.50	1.42	1.00	5.17	4.00
		Did not send to autosegmentation	Forgot	2.09	2.00	1.64	1.00	1.82	2.00	5.83	4.00

	Send T2 MRI to autosegmentation pipeline if applicable	Sent but did not generate contours	Image name not recognized by autosegmentation software	1.73	2.00	1.91	1.00	2.36	2.00	8.00	4.00
			Error in autosegmentation pipeline	2.09	2.00	1.91	1.00	2.45	2.00	8.75	6.00
		Minor Issue: Contours generated but were corrupted	Error in autosegmentation pipeline	1.55	1.00	1.91	1.00	2.36	2.00	7.92	4.00
			Patient anatomy unusual	1.91	2.00	1.91	1.00	2.36	2.00	8.67	5.00
		Major Issue: Contours generated but were corrupted	Error in autosegmentation pipeline	1.55	1.00	2.18	2.00	2.27	2.00	8.42	5.00
			Patient anatomy unusual	1.64	2.00	2.18	2.00	2.27	2.00	9.17	7.00
	Select today's MR and reference MR/CT and contours	Wrong reference image/contours selected	Mistakenly selected wrong image/contours (e.g. chose "for MD" instead of "final" session)	1.55	2.00	2.55	2.00	2.73	3.00	9.92	6.00
			Instructions unclear/had the wrong instructions	1.73	2.00	2.45	2.00	2.82	3.00	11.75	7.00
			Selected the wrong combination of scan and structures (e.g. image from fx1, structures from fx2)	1.09	1.00	2.82	3.00	2.73	3.00	9.08	6.00
		Reference contours not available during 1st fraction	Did not export after plan approval	1.55	2.00	2.18	2.00	1.45	1.00	3.83	4.00
	Run the online setup workflow for the treatment site	Did not run workflow	Forgot	1.18	1.00	2.18	2.00	1.45	1.00	3.25	4.00
			Workflow deleted from MIM	1.18	1.00	2.55	2.00	1.27	1.00	3.42	3.50
		Ran the wrong workflow	Mistakenly selected wrong workflow	1.55	2.00	2.36	2.00	1.55	1.00	5.50	4.00
			Did not know which workflow to use	1.09	1.00	2.36	2.00	1.55	1.00	3.25	2.00
		Workflow is out of date	Proposed changes not updated to MIM yet	1.64	2.00	1.91	1.00	2.45	2.00	7.42	4.50
		Workflow OAR names not match contour names so skips over structure	Name incorrect in the structure set	2.09	2.00	2.45	2.00	2.64	3.00	15.00	10.50
			Structure is new and not recognized yet by workflow	2.00	2.00	2.18	2.00	2.36	3.00	8.92	8.50
		Workflow does not complete	Workflow critical structure (i.e. non-optional) does not exist/is empty	2.00	2.00	2.55	2.00	1.82	2.00	8.08	7.00
	Fuse reference CT/MR and today's MR	Bad fusion	Did not review fusion	1.27	1.00	2.82	3.00	2.18	2.00	7.42	4.50
			Fused to the wrong structure	1.55	2.00	2.64	3.00	2.18	2.00	8.00	8.00
			Unintentional change of fusion after review	1.55	2.00	2.82	3.00	2.91	3.00	12.33	10.00
			Initial rigid CTV fusion (prostate) not good	1.73	2.00	2.64	3.00	2.27	2.00	9.92	8.50
			Initial bone fusion (GI) not good	1.64	1.00	2.55	2.00	2.27	2.00	8.33	8.00
	Record shifts from fusion	Did not record shifts	Forgot	1.64	2.00	1.27	1.00	2.18	2.00	4.00	3.50
	Select deformable or rigid transfer of contours in MIM workflow	Selected the wrong mode	Mistake	1.64	1.00	2.27	2.00	3.09	3.00	10.58	8.50
			Instructions unclear/had the wrong instructions	1.55	1.00	2.09	2.00	3.09	3.00	9.67	7.00
	Import autosegmented contours if applicable (use "add all data")	Did not import autosegmented contours	Forgot	1.45	1.00	2.27	2.00	1.36	1.00	3.67	3.50
	If two copies of the target volume exists from transfer and autosegmentation, remove the worse copy	Did not remove the copy	Forgot	1.82	2.00	2.36	3.00	1.64	2.00	7.50	3.50
			Unaware of this step	1.82	2.00	2.64	3.00	1.82	2.00	11.00	4.00
		Removed the wrong copy	Mistakenly deleted the wrong contour	1.73	2.00	2.64	3.00	2.36	3.00	10.25	6.00
			Did not know which contour to remove	1.45	1.00	2.64	3.00	2.18	2.00	7.58	6.00
	Import subsequent scans as applicable to MIM	Did not import	Forgot	1.36	1.00	2.55	2.00	1.45	1.00	5.25	3.00
			Scan was taken but importing was deemed not needed	1.91	2.00	1.73	1.00	1.27	1.00	4.33	3.00
			Image transfer failed	2.00	2.00	2.64	2.00	1.55	1.00	8.67	6.00
		Imported scan from the wrong day (MIM)	Wrong scan sent to MIM	1.36	1.00	3.18	3.00	1.82	2.00	8.92	7.00
			Selected the wrong scan to bring into the current session	1.45	1.00	3.36	3.00	2.27	2.00	10.75	9.00
	Import subsequent scans as applicable to Monaco	Imported scan from the wrong day (Monaco)	Selected the wrong scan date to bring into the current session	1.45	1.00	4.00	4.00	2.18	2.00	12.83	10.50
2	**Contouring**										
	Clean up external contour to prevent Eclipse transfer error	Did not clean	Forgot	1.91	2.00	1.91	1.00	2.18	2.00	10.00	4.00
		Cleaned but still get transfer error	Did not clean enough slices	1.82	2.00	2.00	1.00	2.00	2.00	7.50	4.00
	Clean up external contour to prevent Eclipse transfer error	Arms not properly included in External contour (if applicable)	Autocontour window did not properly pick up arms	2.18	2.00	2.27	2.00	2.09	2.00	9.33	7.00
	Planner to adjust OARs and CTV as applicable	Minor Issue: Did not adjust	Forgot to adjust OAR	1.55	2.00	3.00	3.00	2.36	2.00	9.08	9.50
			Planner forgot to adjust target	1.36	1.00	2.09	2.00	2.55	2.00	6.42	4.50
		Minor Issue: Adjusted incorrectly	Wrong scan used to adjust contours	1.64	2.00	3.09	3.00	2.64	3.00	12.25	9.00
			Misidentified organ	1.64	2.00	3.00	3.00	2.27	2.00	10.42	7.00
			Planner misidentified target	1.45	1.00	2.27	2.00	2.27	2.00	6.58	6.00
		Major Issue: Did not adjust	Forgot to adjust OAR	1.64	2.00	3.64	4.00	2.36	2.00	13.42	10.50
			Planner forgot to adjust target	1.36	1.00	2.64	3.00	2.27	2.00	8.50	4.50
		Major Issue: Adjusted incorrectly	Wrong scan used to adjust contours	1.64	1.00	3.64	4.00	2.82	3.00	16.67	9.50
			Misidentified organ	1.73	2.00	3.55	4.00	2.27	2.00	14.67	8.50
			Planner misidentified target	1.45	1.00	2.73	3.00	2.36	2.00	9.00	7.00
	Save session as "FX#_forMD"	Saved under a non-obvious name	Unaware of policy	1.27	1.00	1.45	1.00	1.27	1.00	2.67	1.00
			Used wrong field in MIM to change name so it was not updated	1.18	1.00	1.60	1.00	1.27	1.00	2.17	1.50
		Did not save session	Forgot and closed session	1.36	1.00	2.55	2.00	1.36	1.00	4.17	2.50
	MD shares screen and opens MIM session	Did not share screen	Forgot	1.82	2.00	1.55	1.00	1.09	1.00	3.33	2.00
		Opened wrong session	Mistakenly clicked on the wrong session	2.00	2.00	3.27	4.00	2.27	2.00	14.25	16.00
			Instructions unclear/had the wrong instructions	1.45	1.00	3.18	4.00	2.27	2.00	9.92	8.00
		Minor Issue: Edits the wrong structure (i.e. wrong structure selected)	Edited incorrect OAR (e.g. drew large bowel on small bowel)	2.45	2.00	3.18	3.00	2.27	2.00	15.08	15.50
			Edited target but OAR selected (e.g. drew GTV on vessels)	2.09	2.00	3.45	4.00	2.36	2.00	15.17	13.00

	MD reviews CTV, GTV, and OARs and edits as needed		Edited OAR but target selected (e.g. drew vessels on GTV)	2.09	2.00	3.45	4.00	2.36	2.00	15.17	13.00
		Minor Issue: Did not edit structure that required adjustment	Forgot to edit OAR contour	2.36	2.00	3.27	4.00	2.27	2.00	15.33	16.00
			Forgot to edit target contour	1.64	1.00	3.55	4.00	2.64	2.00	15.58	12.00
		Minor Issue: Edited structure incorrectly	Wrong scan used to adjust contours	1.91	2.00	3.45	4.00	2.45	2.00	15.25	16.00
			Misidentified organ	1.64	1.00	3.55	4.00	2.64	3.00	15.42	12.00
			Misidentified target	1.55	1.00	3.64	4.00	3.00	3.00	17.50	12.00
		Major Issue: Edits the wrong structure (i.e. wrong structure selected)	Edited incorrect OAR (e.g. drew large bowel on small bowel)	2.45	2.00	4.18	4.00	2.36	2.00	21.67	24.00
			Edited target but OAR selected (e.g. drew GTV on small bowel contour)	2.00	2.00	4.45	4.00	2.36	2.00	19.33	16.00
			Edited OAR but target selected (e.g. drew small bowel on GTV contour)	2.00	2.00	4.45	5.00	2.36	2.00	19.50	18.00
		Major Issue: Did not edit structure that required adjustment	Forgot to edit OAR contour	2.09	2.00	4.27	4.00	2.36	2.00	19.75	17.00
			Forgot to edit target contour	1.55	1.00	4.45	4.00	3.00	3.00	21.17	15.50
		Major Issue: Edited structure incorrectly	Wrong scan used to adjust contours	1.82	2.00	4.27	4.00	2.45	2.00	16.92	16.00
			Misidentified organ	1.55	1.00	4.55	5.00	2.91	3.00	18.33	15.50
			Misidentified target	1.55	1.00	4.73	5.00	3.18	3.00	21.33	15.50
	MD to check clinical notes if needed	Did not check and it was needed	Forgot	2.27	2.00	4.00	4.00	4.00	5.00	36.75	27.50
		Checked but looked at the wrong reference material	Did not look at newest information	1.73	2.00	4.00	4.00	4.27	5.00	28.50	22.00
			Mistakenly looked at the wrong information	1.36	1.00	4.09	4.00	4.36	5.00	22.92	22.50
	MD to check imaging in PACs if needed	Did not check and it was needed	Forgot	2.27	2.00	3.91	4.00	4.18	5.00	35.00	27.50
		Checked but looked at the wrong reference material	Did not look at newest information	1.64	2.00	3.91	4.00	4.27	5.00	26.00	20.00
			Mistakenly looked at the wrong information	1.36	1.00	3.91	4.00	4.36	5.00	21.25	20.00
	MD saves session as "FX#_MD approved"	Saved under a non-obvious name	Unaware of policy	1.73	1.00	1.09	1.00	1.09	1.00	1.92	1.00
			Used wrong field in MIM to change name so it was not updated	1.27	1.00	1.27	1.00	1.18	1.00	1.58	2.00
	Planner opens MD saved session and shares screen	Opened wrong session	Mistakenly selected the wrong session	1.55	2.00	3.73	4.00	2.18	2.00	10.92	10.00
			Instructions unclear/had the wrong instructions	1.09	1.00	3.64	4.00	2.00	2.00	7.33	7.00
	Run margins workflow specific for the treatment site	Did not run workflow	Forgot	1.09	1.00	3.00	3.00	1.45	1.00	4.58	4.00
			Unaware of policy	1.00	1.00	3.00	3.00	1.45	1.00	3.92	4.00
		Ran the wrong workflow	Mistakenly selected wrong workflow	1.55	1.00	3.09	3.00	1.82	2.00	6.50	7.00
			Workflow was removed from MIM	1.09	1.00	2.91	3.00	1.73	1.00	5.58	3.00
	Enter PTV margins	Inputted wrong values	Mistakenly typed wrong number for target margin	2.27	2.00	4.09	4.00	3.00	3.00	25.75	28.50
			Instructions unclear/had the wrong instructions	1.91	2.00	4.18	4.00	3.09	3.00	24.25	27.00
			Wrong PTV cropping done	2.00	2.00	4.09	4.00	3.09	3.00	23.83	24.00
			PTVopt not created correctly	1.91	2.00	2.64	2.00	2.82	3.00	13.58	11.50
	Enter OAR margins if applicable		Mistakenly typed wrong number for OAR margin	2.18	2.00	3.82	4.00	3.09	3.00	23.58	27.00
			Instructions unclear/had the wrong instructions	1.82	2.00	3.82	4.00	3.18	3.00	20.58	19.00
			Mistakenly type wrong number for OAR cropping	2.27	2.00	3.73	4.00	3.18	3.00	25.00	21.00
	Review margin structures with MD	Did not review	Forgot	1.36	1.00	3.27	3.00	2.09	2.00	8.50	7.00
			Unaware of policy	1.27	1.00	3.36	3.00	2.09	2.00	7.83	7.00
		Reviewed but did not catch error	Did not review thoroughly	1.91	2.00	3.82	4.00	3.09	3.00	20.33	17.00
		Margin structure is blank	did not click "recreate margins"	1.55	2.00	3.64	4.00	2.18	2.00	13.08	8.00
	Review margin structures with MD	For online Monaco only workflow: wrong generation method for contour not discovered (i.e. rigid instead of margin type)	Reference plan incorrect	1.82	2.00	3.45	4.00	2.45	3.00	14.08	15.50
	Contour air if applicable	Did not contour but structure is needed	Unaware of policy	1.55	2.00	3.45	3.00	2.55	2.00	14.17	9.00
			Images did not show that air bubble was in the treatment area (non-optimal sequence to show air)	1.73	2.00	3.36	3.00	3.45	3.00	19.00	15.50
			Images were too old/not updated to reflect air bubble in treatment area	2.09	2.00	3.36	3.00	3.27	3.00	21.42	18.00
		Did not use air scan to contour air	Thought was looking at air scan but wasn't	1.64	2.00	3.27	3.00	2.82	3.00	13.00	14.00
			Not trained properly	1.27	1.00	3.27	3.00	2.91	3.00	11.08	8.50
	Check MM is appropriate	Did not check MM	Forgot	1.73	2.00	2.91	3.00	2.27	3.00	10.42	9.00
			Unaware of policy	1.27	1.00	2.91	3.00	2.36	2.00	9.25	4.50
	Save session as "FX#_Final"	Saved under a non-obvious name	Unaware of policy	1.27	1.00	1.82	1.00	1.55	1.00	3.67	2.00
			Used wrong field in MIM to change name so it was not updated	1.18	1.00	1.91	1.00	1.45	1.00	3.33	2.00
	Export today's MR and contours to offline Monaco	Exported wrong MR/contours	Mistakenly selected the session from the wrong date	1.73	2.00	4.36	5.00	2.73	2.00	17.92	17.00
			Mistakenly selected the "For MD" session from today	1.36	1.00	4.09	4.00	2.82	3.00	13.67	10.50
			Mistakenly selected the "MD approved" session from today	1.36	1.00	3.73	4.00	2.82	3.00	12.42	12.00
		Did not export	Forgot	1.09	1.00	1.82	1.00	1.18	1.00	2.17	1.00
			Transfer fails	1.73	2.00	2.18	1.00	1.64	1.00	6.92	2.50
**3**	**Planning**										
		Import folder changed	IT change but did not update address in Monaco	1.45	1.00	2.55	2.00	2.82	3.00	8.42	4.50

	In offline Monaco, import today's MR and contours	Cannot import	Did not wait for MIM to finish exporting	1.64	2.00	2.27	2.00	1.91	1.00	7.42	3.50
		Import wrong scan/structures	Old scan present in folder	1.64	2.00	4.00	4.00	2.45	3.00	15.08	13.50
			Wrong scan exported	1.64	2.00	4.09	4.00	2.45	3.00	16.17	13.50
	Select reference plan to create adapted plan	Choose wrong reference plan	Mistakenly selected wrong plan	2.09	2.00	2.64	2.00	2.91	3.00	15.92	11.00
			Instructions unclear/had the wrong instructions	2.00	2.00	2.73	2.00	3.09	3.00	15.50	12.00
		Choose ATP instead	Mistakenly selected wrong adaptation method	1.36	1.00	3.18	3.00	2.09	2.00	7.33	7.00
	Save auto fusion in offline Monaco	Auto fusion too far apart to continue	Planner did not move images to be closer	2.18	2.00	1.18	1.00	1.27	1.00	3.00	2.50
	Check ED assignment on adapted plan	Did not check ED assignment	Forgot	1.82	2.00	3.36	3.00	2.82	3.00	17.33	16.00
			Unaware of policy	1.27	1.00	3.27	3.00	2.82	3.00	11.67	7.00
		ED assignment incorrect	Layer of structures incorrect (not external)	2.09	2.00	3.91	4.00	2.91	3.00	22.67	17.00
			Large structure over external making average ED incorrect where there are no contours (e.g. fat, muscles)	1.82	2.00	4.09	4.00	2.55	2.00	19.08	16.00
			Structure requiring override does not have correct ED value not caught (e.g. air not assigned or missing)	1.82	2.00	3.64	3.00	3.09	3.00	19.42	18.00
		ED assignment incorrect	Structure name inconsistent with reference plan so not recognized in adapted plan	2.55	3.00	2.91	3.00	2.64	3.00	18.42	14.00
	Check beam angles/arrangement	Arms in the way of beam	Setup not match simulation	2.09	2.00	3.45	3.00	2.82	3.00	18.33	16.50
	Select optimize shapes	Choose wrong optimization mode	Mistakenly selected wrong mode	1.55	1.00	2.45	2.00	2.18	2.00	6.58	5.50
			Unaware of policy	1.27	1.00	2.45	2.00	2.27	2.00	6.33	5.50
	Check DVH and adjust constraints while optimizing	Did not adjust constraints	Missed editing the objective when optimizing	1.55	1.00	3.00	3.00	2.18	2.00	8.75	8.00
		Minor Issue: Mismatch of structure name causes missing objectives (e.g. bowel that is far)	Changes in Monaco after export to MIM	1.73	2.00	2.27	2.00	2.36	3.00	8.42	7.00
			Changes in MIM causing mismatch with Monaco	1.91	2.00	2.27	2.00	2.36	3.00	8.25	7.00
		Major Issue: Mismatch of structure name causes missing objectives (e.g. PTV or nearby OAR)	Changes in Monaco after export to MIM	1.82	2.00	3.55	4.00	2.27	2.00	14.75	10.00
			Changes in MIM causing mismatch with Monaco	1.91	2.00	3.55	4.00	2.27	2.00	14.67	12.00
		Minor Issue: Optimization objectives incorrect	Changed in reference plan and not noticed	2.09	2.00	2.27	2.00	2.55	3.00	11.33	9.00
			Changed in reference plan and not noticed	2.00	2.00	3.18	3.00	2.55	3.00	16.08	14.00
		Minor Issue: Mismatch of structure name causes missing DVH criteria	Changes in Monaco after export to MIM	1.55	1.00	2.55	2.00	2.82	3.00	10.67	8.50
			Changes in MIM causing mismatch with Monaco	1.91	2.00	2.55	2.00	2.82	3.00	13.17	10.00
		Major Issue: Mismatch of structure name causes missing DVH criteria	Changes in Monaco after export to MIM	1.55	1.00	3.36	4.00	2.55	3.00	14.92	11.00
			Changes in MIM causing mismatch with Monaco	2.00	2.00	3.45	4.00	2.73	3.00	18.67	18.00
		Minor Issue: DVH template has incorrect constraints	Changed in reference plan and not noticed	2.00	2.00	3.09	3.00	3.18	3.00	18.50	15.00
			Changed in reference plan and not noticed	2.00	2.00	4.09	4.00	3.18	3.00	25.17	21.00
	Acquire verification 3D scan when optimization is at "starting segmentation"	Did not acquire verification scan	Forgot	1.25	1.00	3.25	3.50	1.42	1.00	5.58	4.00
			Unaware of policy	1.17	1.00	3.25	3.50	1.67	1.00	6.25	4.00
		Wrong sequence taken for verification scan	Mistakenly selected wrong sequence	1.42	1.00	2.75	2.50	2.00	2.00	8.58	4.50
			Unaware of policy	1.17	1.00	2.75	2.50	2.08	2.00	6.33	5.50
	Planner to go to Sequencer computer and share screen	Did not share screen	Forgot	1.92	2.00	1.50	1.00	1.08	1.00	3.08	2.50
	In online Monaco, click on the "open" icon which will open the saved plan from previous steps in the planning tab	Clicked "open" in Online Monaco before plan was saved	Unaware of policy	1.55	1.00	2.00	2.00	2.09	2.00	7.50	4.00
		The "open" option not clickable in Online Monaco	UID bug in Monaco where UID is blank and system does not allow user to open plan	1.73	2.00	2.18	1.00	1.64	1.00	5.33	2.50
			Forgot to save plan before closing Offline Monaco	1.55	1.00	2.36	1.00	1.27	1.00	3.17	3.00
		Dose missing when opening plan from offline Monaco	Objective was changed just as the plan finished calculation in offline Monaco	1.91	2.00	1.82	1.00	1.91	1.00	5.75	4.00
	Update MM	Did not update MM	Forgot to update MM	2.09	2.00	3.18	3.00	2.91	3.00	16.58	15.50
	Recheck ED in online Monaco	Did not recheck ED	Forgot to recheck but checked in offline Monaco	2.18	2.00	2.64	3.00	3.00	3.00	18.83	13.00
			Forgot to check in both offline and online Monaco	1.55	1.00	3.18	3.00	2.73	3.00	12.33	12.50
**4**	**Verification and Plan Approval**										
	Send verification MR to online Monaco	Sent images before plan was opened in Monaco	Unaware of policy	1.42	1.00	2.25	2.00	1.83	2.00	6.17	4.00
		Wrong images sent to Monaco	Wrong sequence from today exported from MARLIN	1.58	2.00	3.33	4.00	2.92	3.00	15.25	13.50
			Wrong image date exported from MARLIN	1.25	1.00	3.50	4.00	2.75	2.50	11.67	10.00
	Scroll and review contours and dose on	Missed small patient shift	Did not perform ATP when it was needed	1.75	2.00	2.92	3.00	2.92	3.00	13.08	12.00

	Scroll and review contours and dose on verification MR with MD	Missed large patient shift	Did not redo ATS when it was needed	1.92	2.00	4.00	4.00	2.75	2.50	20.50	16.00
		Wrong image used for review	Looked at wrong image (i.e. secondary instead of primary)	2.08	2.00	4.17	4.00	2.92	3.00	24.83	24.00
	If needed, perform second adaption	ATP performed when ATS-lite should have been used due to magnitude of shift	Did not know to use "ATS-lite"	2.00	2.00	3.25	3.00	2.75	3.00	18.33	16.00
		ATS-lite performed incorrectly	Not all structures set to rigid (except external)	2.17	2.00	2.92	2.50	2.50	2.50	15.67	12.00
		Organ/tumor changed significantly such that it is not accurately represented by contour and did ATP instead of 2nd ATS (i.e. bowel moved)	Missed in review of verification scan	2.00	2.00	3.91	4.00	3.00	3.00	20.92	18.00
		Exported but did not receive data	Mistakenly selected wrong destination	1.91	2.00	1.45	1.00	2.00	2.00	5.83	2.50
			Address changed but not updated in Monaco	1.45	1.00	2.00	1.00	2.82	3.00	7.75	5.00
		Did not send image to IMU	Unaware of policy	1.18	1.00	1.91	2.00	1.64	1.00	3.58	2.00
		Did not send structures	Unaware of policy	1.18	1.00	1.91	2.00	1.64	1.00	3.58	2.00
		Did not send total plan	Unaware of policy	1.18	1.00	1.91	2.00	1.64	1.00	3.58	2.00
		Did not send total plan dose	Unaware of policy	1.18	1.00	1.91	2.00	1.64	1.00	3.58	2.00
		Did not sent individual beam dose	Unaware of policy	1.18	1.00	1.91	2.00	1.64	1.00	3.58	2.00
	Select "DICOM export" and send to only Total Plan to "LinacView"	Exported but did not receive data	Mistakenly selected wrong destination	1.64	2.00	1.73	1.00	1.55	1.00	4.67	2.00
			Address changed but not updated in Monaco	1.09	1.00	2.18	2.00	2.64	3.00	6.42	3.50
		Did not send to LinacView	Forgot	1.55	1.00	1.82	1.00	1.73	1.00	4.58	3.50
		Correct plan sent but LinacView not adequately prepared	Old plan still exists in LinacView	2.00	2.00	1.73	1.00	2.18	2.00	6.83	4.00
	MD approves plan in online Monaco via "request control" function on Webex	Request control function unavailable	Webex issue not allowing this function	2.00	2.00	2.25	1.50	1.58	1.00	8.25	4.00
	Planner closes Monaco	Did not close Monaco	Forgot	1.92	2.00	1.50	1.00	1.58	1.00	13.00	2.00
5	**Plan Review and Treatment**										
	Choose correct phantom for lung IMU	Did not use correct phantom for IMU	Unaware of policy	1.70	2.00	2.20	2.00	1.90	2.00	5.67	7.00
	Run IMU	IMU calculation point in the wrong place	Did not move to appropriate location	2.20	2.00	2.00	2.00	2.20	2.50	8.92	6.50
			Reference point was not called "IMU"	2.10	2.00	1.80	1.50	2.00	2.00	6.67	6.00
	When ~500MU is left, take post treatment MR if needed	Started post treatment scan too early, patient moves so it is not caught on MM	Unaware of policy	1.91	2.00	2.82	3.00	3.27	4.00	18.75	14.00
		Did not take post treatment scan	Forgot	1.36	1.00	2.64	3.00	1.82	2.00	6.08	3.50
			Unaware of policy	1.00	1.00	2.64	3.00	1.73	2.00	4.17	3.50
6	**Post Treatment**										
	Plan checker saves results of Sanity Check, PIC and IMU	Did not save results	Forgot	1.40	1.00	1.20	1.00	1.10	1.00	1.58	1.00
	Compile report with today's plan + IMU and planner and checker signs	Did not compile report	Forgot	1.80	2.00	1.20	1.00	1.10	1.00	1.92	2.00
		Did not sign complied report	Forgot	2.10	2.00	1.30	1.00	1.30	1.00	3.08	2.50
	Planner and plan checker makes appropriate notes in Online Adaption Form	Did not fill out form	Forgot	2.30	2.00	2.30	2.50	2.30	2.00	11.67	6.00
		Form filled out incorrectly	Forgot to include relevant notes	2.00	2.00	2.20	2.00	2.80	3.00	10.17	4.00
	In ARIA: bill patient treatment	Did not bill	Forgot	2.18	2.00	1.55	1.00	1.64	2.00	6.42	3.50
			Billed incorrect codes	1.73	2.00	1.55	1.00	2.36	2.00	6.33	3.50
	Adds "MR Adaptive Procedure Note" in ARIA and add fraction number	Incorrect fraction number	Mistakenly filled out with incorrect fraction number	2.00	2.00	1.18	1.00	2.18	2.00	5.50	4.00
	Assign "Approve MRL procedure" task to MD	Did not add task	Forgot	1.90	2.00	1.20	1.00	1.90	1.50	3.75	2.50
		Added task but to the wrong MD	Mistake	1.90	2.00	1.20	1.00	2.00	2.00	3.67	3.00
	MD to approve "MR Adaptative Procedure Note"	Did not approve	Forgot	2.00	2.00	1.20	1.00	1.70	2.00	3.42	3.00
	Send post MR to offline Monaco	Did not send	Forgot	2.09	2.00	1.73	1.00	1.55	1.00	7.42	3.00
			Sent the wrong scan	1.82	2.00	1.91	2.00	2.36	2.00	7.67	5.00
	Planner imports post MR in offline Monaco	Did not import	Forgot	1.64	1.00	2.09	2.00	1.91	2.00	6.58	3.50
	In Offline Monaco: review contours from treated plan on post MR	Incomplete review of the post MR	Reviewed wrong image	1.82	2.00	2.91	3.00	3.00	3.00	13.83	12.00
			Reviewed and thought image looked ok but there was a large change/shift not noticed by the planner	1.45	1.00	3.36	3.00	3.36	4.00	14.42	15.50
	Notify MD if large shift has occurred	Did not notify MD	Forgot	1.73	1.00	3.55	4.00	3.09	3.00	20.83	12.00
	**END**										
