# Supplementary material for: Risk analysis of the Unity 1.5T MR‐Linac adapt‐to‐shape workflow
Source: J Appl Clin Med Phys. 2025 Apr 16;26(7):e70095. doi: 10.1002/acm2.70095 (PMC12256694; doi:10.1002/acm2.70095)
Supplement: Supplementary file 3 — Supporting Information [file ACM2-26-e70095-s003.pdf]

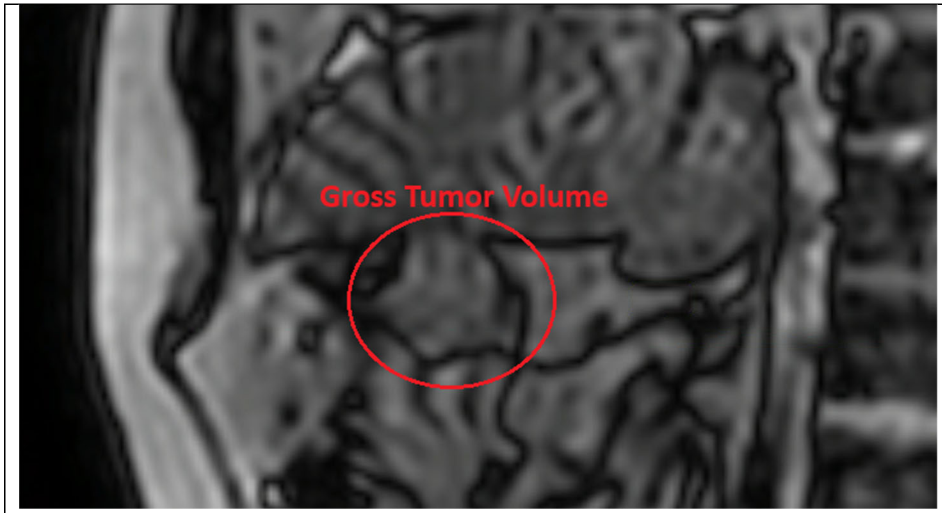

1. Gross tumor volume shown in red circle

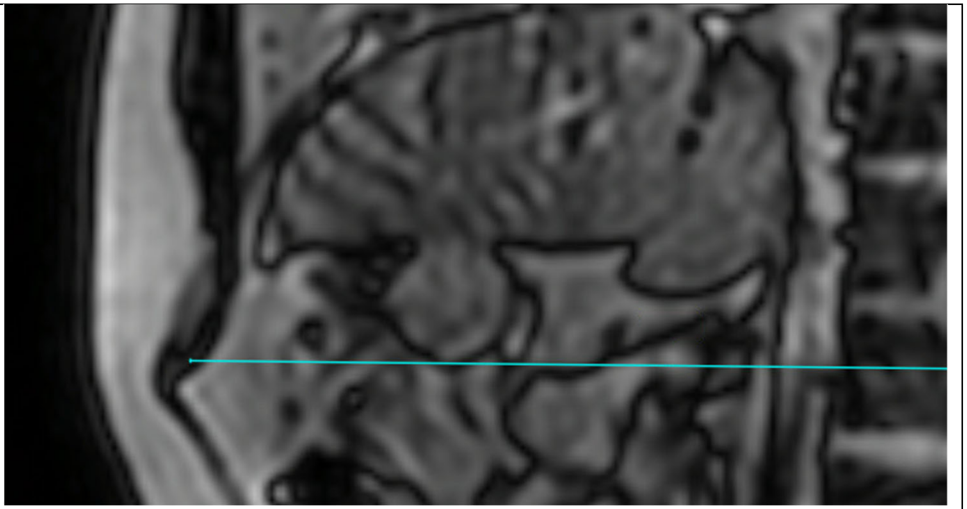

2. Inferior extent of gross tumor at inhale shown by blue line

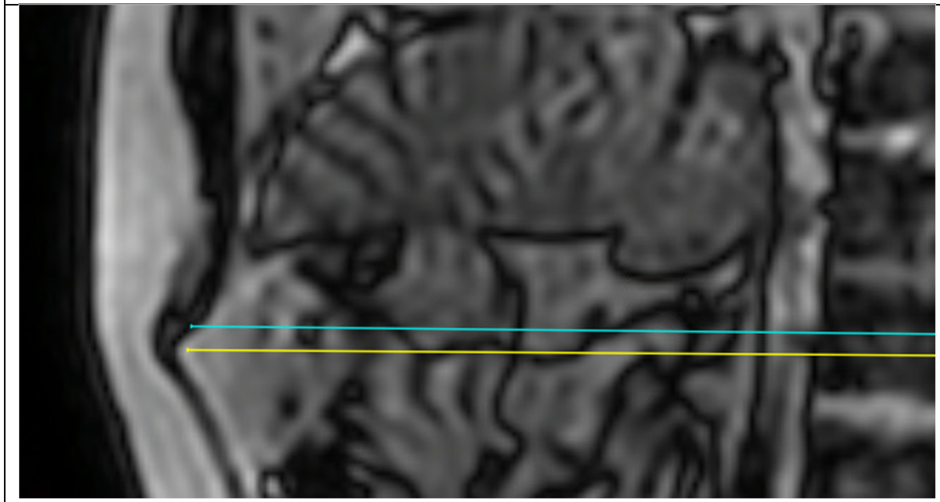

3. Inferior extent of gross tumor at exhale shown by yellow line

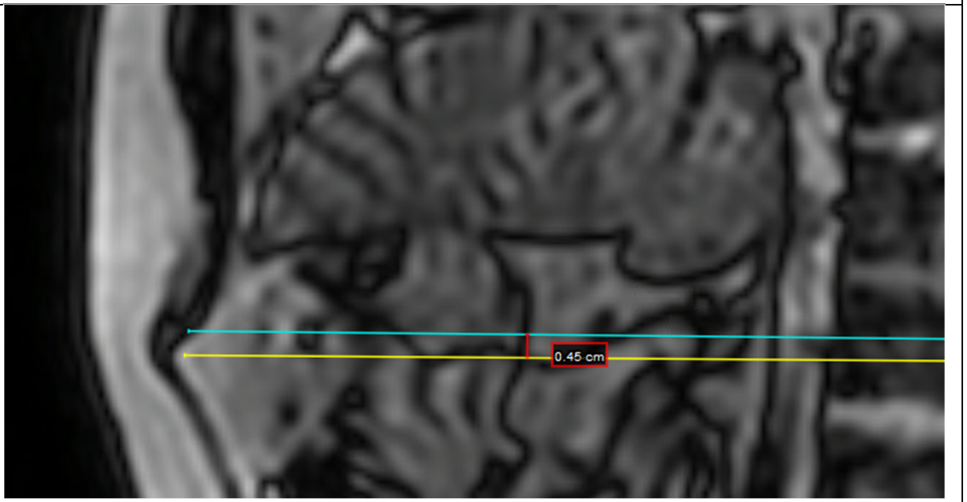

4. Measurement of the extent of motion (0.45cm difference)

Example of the evaluation of gross tumor motion with the use of a compression belt at simulation. Evaluation is done using a cine-MRI; screen shots of the full inhale and full exhale phases are shown to illustrate how tumor motion is evaluated.
